# FastAnnotator- an efficient transcript annotation web tool

**DOI:** 10.1186/1471-2164-13-S7-S9

**Published:** 2012-12-07

**Authors:** Ting-Wen Chen, Ruei-Chi Richie Gan, Timothy H Wu, Po-Jung Huang, Cheng-Yang Lee, Yi-Ywan M Chen, Che-Chun Chen, Petrus Tang

**Affiliations:** 1 Molecular Medicine Research Center, Chang Gung University, Taoyuan, Taiwan; 2 Bioinformatics Center, Chang Gung University, Taoyuan, Taiwan; 3 Department of Biological Science and Technology, National Chiao Tung University, Hsinchu, Taiwan; 4 Institute of Biomedical Informatics, National Yang-Ming University, Taipei, Taiwan; 5 Graduate Institute of Biomedical Sciences, Chang Gung University, Taoyuan, Taiwan; 6 Department of Microbiology and Immunology, Chang Gung University, Taoyuan, Taiwan; 7 Department of Aquatic Biosciences, National Chiayi University Chiayi, Taiwan

## Abstract

**Background:**

Recent developments in high-throughput sequencing (HTS) technologies have made it feasible to sequence the complete transcriptomes of non-model organisms or metatranscriptomes from environmental samples. The challenge after generating hundreds of millions of sequences is to annotate these transcripts and classify the transcripts based on their putative functions. Because many biological scientists lack the knowledge to install Linux-based software packages or maintain databases used for transcript annotation, we developed an automatic annotation tool with an easy-to-use interface.

**Methods:**

To elucidate the potential functions of gene transcripts, we integrated well-established annotation tools: Blast2GO, PRIAM and RPS BLAST in a web-based service, FastAnnotator, which can assign Gene Ontology (GO) terms, Enzyme Commission numbers (EC numbers) and functional domains to query sequences.

**Results:**

Using six transcriptome sequence datasets as examples, we demonstrated the ability of FastAnnotator to assign functional annotations. FastAnnotator annotated 88.1% and 81.3% of the transcripts from the well-studied organisms *Caenorhabditis elegans *and *Streptococcus parasanguinis*, respectively. Furthermore, FastAnnotator annotated 62.9%, 20.4%, 53.1% and 42.0% of the sequences from the transcriptomes of sweet potato, clam, amoeba, and *Trichomonas vaginalis*, respectively, which lack reference genomes. We demonstrated that FastAnnotator can complete the annotation process in a reasonable amount of time and is suitable for the annotation of transcriptomes from model organisms or organisms for which annotated reference genomes are not avaiable.

**Conclusions:**

The sequencing process no longer represents the bottleneck in the study of genomics, and automatic annotation tools have become invaluable as the annotation procedure has become the limiting step. We present FastAnnotator, which was an automated annotation web tool designed to efficiently annotate sequences with their gene functions, enzyme functions or domains. FastAnnotator is useful in transcriptome studies and especially for those focusing on non-model organisms or metatranscriptomes. FastAnnotator does not require local installation and is freely available at http://fastannotator.cgu.edu.tw.

## Background

As sequencing technologies have improved, transcriptome sequencing (RNA-Seq) or whole-genome sequencing have become faster and cheaper than ever before. In addition, sequencing projects addressing non-model organisms or environmental samples (metagenomics) have now become feasible and affordable. For example, the sequencing of relatively unexplored organisms, such as the human gut microbiome or the transcriptome of clams or butterflies, has been accomplished [1-3]. However, sequencing is only the first step in the process of understanding the biology underlying the obtained sequences. Bioinformatics analyses performed after sequencing, including assembly, functional annotation and classification, are becoming increasingly important. Several annotation pipelines have been developed, such as CycADS, PRIAM and Blast2GO [4-6], which can provide putative functions for a transcript based on sequence similarity to known genes. Although these tools are useful for understanding the newly explored sequences, they usually contain many manual steps and often are not easy to implement for biologists who are unfamiliar with the command line inputs. Web tools have been developed for the annotation of expressed sequence tags (EST), such as ESTAnnotator, ESTpass, and ESTExplorer [7-10]. These pipelines are specifically designed for EST analyses that include the cleaning, assembly and clustering and functional annotation of ESTs. However, we found that the online Uniform Resource Locators (URLs) for ESTpass and ESTAnnotator are no longer functional for ESTpass and ESTAnnotator, and ESTExplorer cannot simultaneously process more than ten thousand contigs. Thus the development of a web server that can provide an automatic annotation pipeline for contigs derived from RNA-Seq data with a user-friendly interface would be beneficial to the research community.

Functional annotation strategies are typically based on previously identified protein functions. Many protein function databases, such as BRENDA, Enzyme and Amigo, have been established as collections based on the functions of enzymes or genes [11-13]. These databases categorize protein functions into structured groups as either Enzyme Commission numbers (EC numbers) or Gene Ontology (GO) terms. EC numbers are assigned to enzymes according to the chemical reactions they catalyze [14], whereas GO terms include information on the molecular function, cellular component and biological processes of genes and can be used to describe the biological function of proteins [15]. These protein function or enzyme function databases document well-studied and well-annotated biological functions and provide resources for the annotation of newly sequenced genes. The majority of established annotation methods utilize these functional annotation systems and transfer functional annotations among sequences based on sequence similarity or pattern searches. For example, the CycADS pipeline integrates multiple annotation tools and databases [4]; PRIAM identifies enzyme functions based on profiles constructed from known enzymes [6]; and Blast2GO annotates gene functions based on a combinations of various annotation methods [5]. The strategy of annotation transfer has been shown to work well and is frequently employed.

Herein, we present an automatic, efficient and easy-to-use web-based annotation tool named FastAnnotator. Given that many tools have been proposed to annotate sequences based on previously established knowledge, we chose to integrate several well-established and popular tools to construct this service. FastAnnotator utilizes Blast2GO and PRIAM to identify GO terms and EC numbers for transcript sequences. Blast2GO has a high annotation accuracy (65-70%) [16], and PRIAM provides an enzyme profile database that was updated in October 2011 [6]. As certain assembled sequences identified using RNA-Seq may not cover full-length coding regions, we also included a domain search in FastAnnotator. In addition to the possibility of identifying domains based on partial transcript sequences, domain searching also enables FastAnnotator to identify possible functions for sequences that show a significant level of divergence. This feature was included because sequences may evolve to become dissimilar, although the functional domain regions are likely to be conserved through evolution under functional constraints. It has also been shown that proteins having the same domain composition are homologs and likely have the same function [17-19]. Therefore, domain annotation in FastAnnotator can not only provide an annotation for a partially sequenced transcript but also improve the annotation performance for sequences that are highly divergent from existing database sequences. This property is particularly useful for metatranscriptomic analyses or samples from organisms that are distantly related to model organisms.

In summary, we have developed the FastAnnotator pipeline to provide automatic annotation of nucleotide sequences via a web interface. Users can begin their annotation by uploading their sequences to the FastAnnotator website. FastAnnotator then assigns possible functions and identified domains in those input query sequences based on sequence similarity and pattern searches. The output of FastAnnotator includes the best hits in the NCBI non-redundant database, GO terms, EC numbers, and domain identities [20,21]. Users can explore the output on the website, access other external functional databases (BRENDA, Amigo or Pfam) for detailed functional descriptions and simply download the output for further analysis. As a web service specifically developed for ease-of-use annotations, the use of FastAnnotator does not demand any knowledge of the command line. Moreover, FastAnnotator is free, efficient and user-friendly. Relative to existing annotation pipelines, FastAnnotator is much more convenient because it does not require installation or tedious manual steps and allows the user to analyze a large number of sequences with a single click. FastAnnotator is now available at http://fastannotator.cgu.edu.tw/.

## Implementation

### Flowchart of the FastAnnotator pipeline

**Figure 1:**
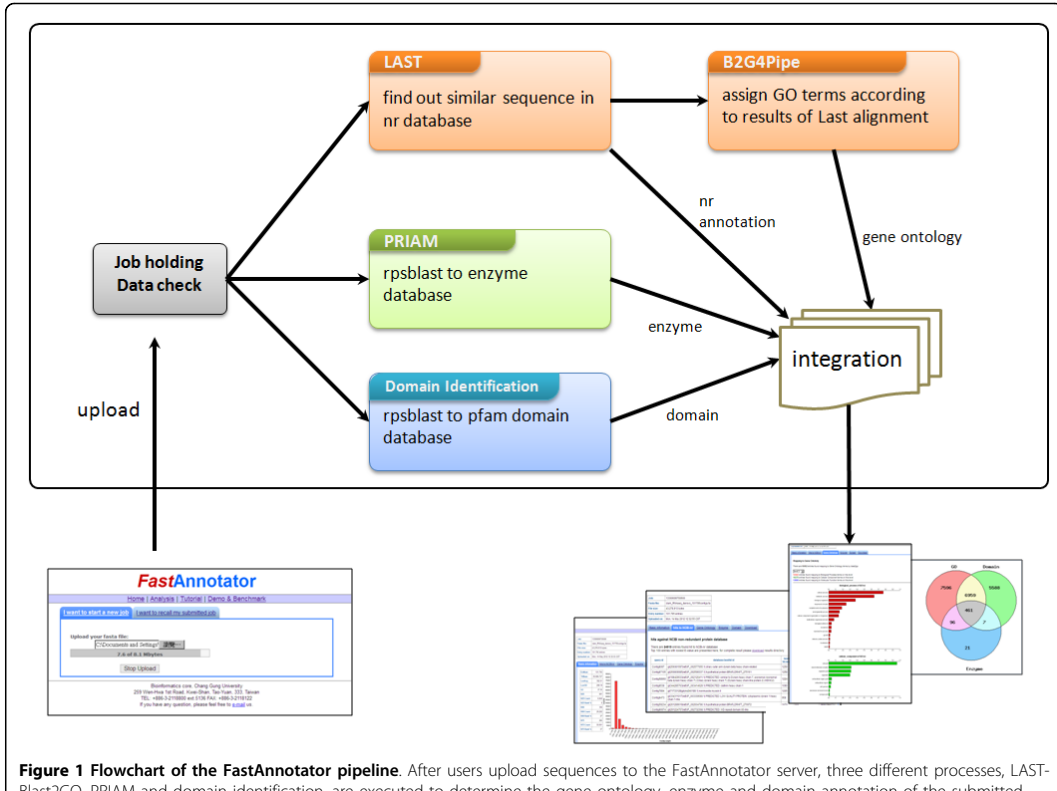
**Flowchart of the FastAnnotator pipeline**. After users upload sequences to the FastAnnotator server, three different processes, LAST-Blast2GO, PRIAM and domain identification, are executed to determine the gene ontology, enzyme and domain annotation of the submitted sequences. We implemented these three processes in parallel to accelerate the annotation procedure. After all of these annotation programs are completed, FastAnnotator presents GO terms, the best hits in the nr database, enzyme annotations and domain annotations together with a statistical report on the website. In addition to exploring the annotation results online, users can also download all of the annotation results as a zip file.

The annotation process in FastAnnotator consists of four main parts: finding the best hit in the NCBI non-redundant database, assignment of GO terms, identification of enzymes, and identification of domains. The assignment of the GO terms requires the result from LAST searching against the NCBI non-redundant database, and these four main steps can therefore be divided into three independent modules: GO term assignment, enzyme identification, and domain identification (Figure 1). FastAnnotator runs these three steps in parallel to accelerate the annotation procedure and also calculates the basic statistics of the input sequences and provides a statistical report in the results.

### The identification of GO terms

LAST [22-24] and Blast2GO (B2G4Pipe) [5] were used together to identify GO annotations for the query sequences. We downloaded the non-redundant protein sequences from the NCBI database [21] and constructed a local database for Blast2GO. In FastAnnotator, LAST is used to find the similar sequences within the non-redundant protein sequence database. The output of LAST is then transformed into the equivalent BLASTX XML output format, which is the required input file format for Blast2GO. We used the statistical parameters λ and κ provided by the LAST output result to derive the bit score based on the following definition [25]:

$$\text{S' = }\frac{\lambda \;\text{S}\;\text{ - }\;\text{In}\;\text{κ}}{\text{In}\;\;\text{2}}$$

The alignment result generated by LAST is then used as an input to Blast2GO. The final assignments of GO terms are extracted and presented in a table on the website, which is made available for download.

### The identification of domains

We downloaded the standalone BLAST+ (v2.2.25) program from NCBI [26] and used as database the 13,672 domain models (Pfam v26) from the Conserved Domains Database (CDD) [19,20]. FastAnnotator applies the rpstblastn to identify domains in the query nucleotide sequences by searching against the preformatted domain database with mostly default parameters except the expectation value (e-value) which is set to be less than 0.01, and the hit aligned length which is longer than 50% of the domain PSSM [27]. After the domains of the query sequences are identified, FastAnnotator calculates the length coverage and presents the percentage of coverage for the domain in the report table.

### The identification of EC numbers

FastAnnotator utilizes PRIAM [6] to identify potential enzyme functions. PRIAM can detect specific enzymes patterns and annotate these enzymes with EC numbers. The latest version of the enzyme profiles (released on 19 Oct, 2011) was downloaded from the PRIAM website. The nucleotide sequence inputs were translated into protein sequences in six frames using Transeq, a tool a part of the European Molecular Biology Open Software Suite (EMBOSS) [28]. These translated protein sequences are then used as inputs to search against the enzyme profile database. FastAnnotator identifies transcripts that may act as enzymes and presents the transcripts together with EC numbers in the output table.

## Results and discussion

**Figure 2:**
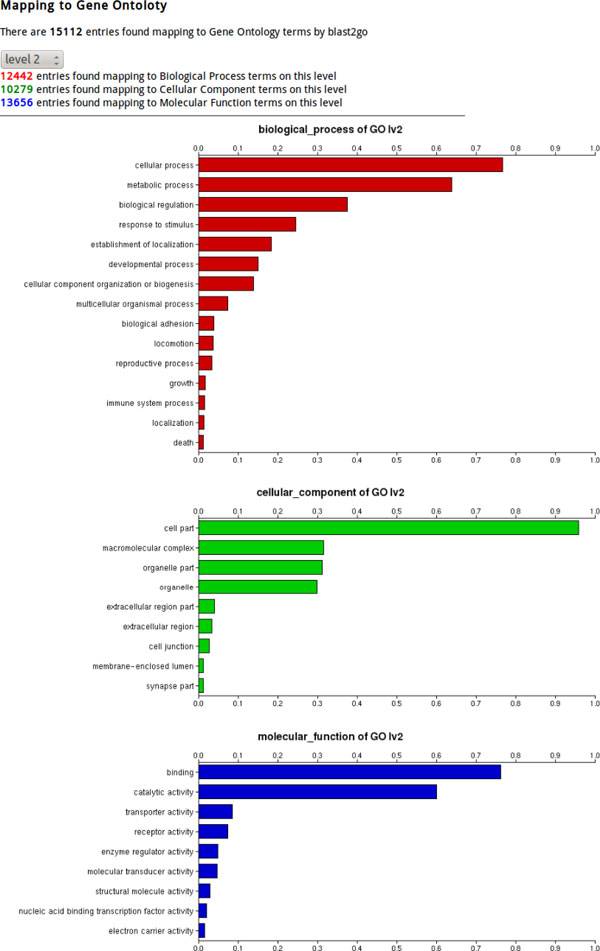
**Partial result of a GO annotation using FastAnnotator**. The GO annotation results include horizontal bar charts in addition to a table of sequences and corresponding GO annotations. These three horizontal bar charts present the distribution of GO terms categorized as biological process, cellular components or molecular functions, which represent the three main categories for GO annotation. Because the GO terms have a hierarchical structure, FastAnnotator provides views of the distribution of GO terms at different levels. Users can select the level and obtain the overall distribution of the GO terms in real-time. For example, level 1 presents the roots (e.g., biological process, cellular components and molecular functions), and level 3 presents the GO terms that are 2 edges away from the roots.

The FastAnnotator website allows users to upload *de novo *assembled sequences from RNA-Seq dataset through the upload page (shown in Figure 1). Only FASTA files of less than 100 megabytes are able to be uploaded to the server. After a successful upload, a unique job ID is provided as an identifier to start the annotation pipeline. All annotation results can be inspected over the Internet or downloaded in a compressed file for further analysis. The report consists of five sections. The first section is a histogram of the length distribution of the input sequences as well as a list of other statistics, including the average length, total number of bases, and the N50 value. The second section lists the best hits for all sequences against the NCBI non-redundant database. To avoid overloading the browser, only the top 100 entries are shown on the website results page as a demonstration, although users can download all of the best hits from the download link. The third section contains the results of the functional annotation (GO terms). GO term annotations are grouped according to biological processes, cellular components and molecular functions and are plotted in three separate horizontal bar charts; an example is shown in Figure 2. To better represent the overall distribution of functional annotations, additional spread-out or clustered views are provided based on different filtering level of GO terms. For example, when level 3 is chosen, all annotated GO terms are traced back to the root until reaching the nodes that were 2 edges away from the root (the 3^rd ^node) on the GO acyclic graph. Users can then choose among different level of GO terms to get a better biological interpretation. The fourth section presents the enzyme annotation, which lists all enzyme hits and their corresponding query sequences. The last section contains a list of domain identifications, and again, only 100 entries are shown to avoiding overloading the browser. All of these annotations can be downloaded as a compressed zip file. Annotation results for each job will be retained on the FastAnnotator server for two weeks after the job is finished, and users can retrieve the finished job using the job IDs provided by the server.

To demonstrate the performance and speed of FastAnnotator, we ran it on several transcriptome sequence datasets from different organisms. We were especially interested in how FastAnnotator could assist in the annotation of non-model organisms. The unpublished RNA-Seq dataset from a clam (*Meretrix meretrix*) was used as an example. This transcriptome included 22,129,105 cDNA sequence reads generated using an Illumina Genome Analyzer, and the average read length was 80 base pairs. A reference genome for the clam is currently not available, and thus, the reads were assembled *de novo *into 101,795 contigs using the software package CLC Genomics Workbench (CLC bio, Denmark). Using these contigs as the input, FastAnnotator required approximately 16 hours to finish the annotation. The statistical output showed that the N50 of these contigs was 390 nucleotides and the majority of these assembled contigs (73,878 contigs) ranged from 200-399 base pairs. This large number of short contigs may have resulted from a low level of coverage, and we believe that many of these contigs were actually partial transcript sequences. Of these contigs, 24,919 were found to be similar to sequences in the NCBI non-redundant protein database; 15,112 were assigned at least one Gene Ontology term; 13,015 were found to contain at least one domain; and 585 were assigned an enzyme annotation. Among all of the contigs, 20.4 % of contigs were annotated, and the annotation rate was even higher (26.4%) if we only considered contigs longer than 250 base pairs. These contig annotation rate was slightly higher than that reported in previous studies [1,29], the majority of which were based on domain identification. A further comparison of our annotation results with a recently published clam transcriptomic analysis by Huan et al. [1] showed that our annotation results for the clam transcriptome were similar.

We were interested in the performance of FastAnnotator for different organisms from different evolutionary lineages. We tested FastAnnotator using six transcriptome datasets, which includes data from plants, invertebrates, protists and prokaryotes (Table 1). All of these transcriptomes were derived from the *de novo *assembly from NGS reads with the exception of the amoeba transcriptome, which was derived from EST data. Reference genomes were available for *C. elegans *and *S. parasanguinis *[30], and the highest sequence annotation percentages were obtained for these organisms. Given the high accuracy obtained using Blast2GO, which was used in the FastAnnotator annotation, these annotation results can be considered highly reliable. For the non-model organisms, FastAnnotator was able to annotate approximately half of the input sequences in three out of the four cases. We found the annotation rate was much lower than half in clam transcriptome (20.4%). Meanwhile we found the percentage of annotated contigs in our clam transcriptome was similar to those reported in previous clam studies (12.2% and 20.7% for the clam *M. meretrix *transcriptome and an Agamaki clam EST study, respectively [1,29]) and the dataset used here was composed with numerous of partial transcripts. Thus we conclude that FastAnnotator is a suitable annotation tool for non-model organisms.

**Table 1:** FastAnnotator results for five different organisms

Organism	# of entries (total base)	Processing time	% of sequences with best hit	% of annotated sequence^*^
*C. elegans*^+^	4,456 (1,339,253)	1.25 h	97.8%	88.1%
Bacteria example^+^	1,219 (423,067)	21 min	94.7%	81.3%
Plant example^+^	39,914 (24,168,315)	16 h	77.0%	62.9%
Clam^+^	101,795 (38,886,727)	15.5 h	24.5%	20.4%
Amoeba EST	13,814 (11,055,101)	3.5 h	68.4%	53.1%
*Trichomonas vaginalis*^+^	35,989 (26,716,478)	11 h	93.7%	42.0%

^+^RNA-Seq denovo assembly result

*Percentages of sequences had been annotated with GO, enzyme or domains.

GO annotation is the most commonly used and well-established functional annotation scheme. To identify GO terms for the input sequences, FastAnnotator incorporates the Blast2GO pipeline, which is one of the most widely used annotation tools and claims to have an annotation accuracy of 65-70% [16]. However, we made a small modification to the Blast2GO pipeline by replacing BLASTX with LAST, as LAST is significantly faster and is capable of detecting evolutionarily conserved regions [23]. We also used the example file provided by the B2G4Pipe package to benchmark the computation time and comparing output results of BLASTX and LAST on the same machine (IBM X3850 with four Xeon E7540 2.0GHz CPUs and 128G RAM). We found that for this file of 10 nucleotide sequences, BLASTX took 5 times longer to search against the non-redundant database comparing to LAST. The resulting 30 annotations reported by each tool differed in only 2 cases. A closer inspection of the results revealed that the differences were minor and could be explained by the parent and child relationship within the GO acyclic graph. Therefore, we concluded that replacing BLASTX with LAST resulted in a minimal difference in the annotation while providing a substantial improvement in the computational speed.

Regarding the detection of enzymes, FastAnnotator annotates enzyme functions based on the database of 2,844 EC numbers included in the most recent PRIAM release. According to Integrated relational Enzyme database (IntEnz) release 76, there were 4,812 active EC numbers [30]. Furthermore, it is well-known that more than one-third of the enzyme activities with EC numbers are so-called orphan enzyme activities, which are not associated with a protein or gene sequences [32-34]. Approximately 50% of the orphan enzyme activities are limited to only one species or closely related organisms, which implies that orphan enzyme activities may be limited to certain organisms that remain to be fully explored [35]. Due to these limitations, current annotation tools that are based on sequence similarity searches are restricted to detecting only certain enzyme activities. Consequently, FastAnnotator may overlook certain enzyme functional annotations, especially if those functions are restricted to particular organisms. In our clam transcriptome, for example, only 585 contigs were identified as enzymes. Because it has been estimated that approximately 18-29% of genes encode enzymes in eukaryotes [36], it is very likely that this level of enzyme annotation was an underestimate, which may have occurred due to the fact that the majority of the contigs are only partial transcripts in our clam example and because the enzyme database used in FastAnnotator was imcomplete. FastAnnotator may provide improved annotations in the future after additional enzyme functions and associated sequences are identified and included in the enzyme databases.

## Conclusions

As sequencing technologies improve and decrease in cost, an increasing number of sequences can be generated. However, the annotation of these sequences, especially for those generated from unfamiliar biological samples, becomes an important issue. In this project, we developed FastAnnotator, an automatic annotation web tool, which integrates several well-developed annotation tools together to provide annotations for query sequences. FastAnnotator allows users to assign protein functions, cellular location, enzyme activity, and function domains to query sequences through an easy-to-use interface. By adopting a different sequence search program, LAST, and including domain identification, it is capable of efficiently annotating sequences and is suitable for annotation of sequences derived from less well-studied organisms or environmental samples. In summary, we present a web-based annotation tool, FastAnnotator, which should be helpful in transcriptome studies, particularly metatranscriptome or non-model organism studies.

## Availability and requirements

Project name: FastAnnotator

Project home page: http://fastannotator.cgu.edu.tw

Hardware specifications: IBM X3850 with four Xeon E7540 2.0GHz CPUs and 128G RAM

Operating system(s): CentOS Release 5.7 with Linux kernel 2.6.18

Programming language: Python and Perl for pipeline building, PHP for website interface.

Browser requirements: The website can be access by popular web browser with Javascript enabled include Mozilla Firefox, Google Chrome and Microsoft Internet Explorer.

## Competing interests

The authors declare that they have no competing interests.

## Authors' contributions

TWC and THW built the pipeline. RCRG designed and constructed the website. TWC and RCRG wrote the manuscript together. PJH and CYL maintain the system. YMC and CCC performed RNA-Seq of *Streptococcus parasanguinis *and clam. PT and CCC supervised and revised the manuscript.
